# A simple method for rapid cloning of complete herpesvirus genomes

**DOI:** 10.1016/j.crmeth.2024.100696

**Published:** 2024-01-23

**Authors:** Jan Knickmann, Laura Staliunaite, Olha Puhach, Eleonore Ostermann, Thomas Günther, Jenna Nichols, Michael A. Jarvis, Sebastian Voigt, Adam Grundhoff, Andrew J. Davison, Wolfram Brune

**Affiliations:** 1 Leibniz Institute of Virology (LIV), Hamburg, Germany; 2 MRC-University of Glasgow Centre for Virus Research, Glasgow, UK; 3 School of Biomedical Sciences, University of Plymouth, Plymouth, UK; 4 The Vaccine Group Ltd., Plymouth, UK; 5 Institute for Virology, University Hospital Essen, Essen, Germany

**Keywords:** herpesvirus, DNA virus, BAC, YAC, YCp, recombination, yeast, RCMV, KSHV, HHV-8

## Abstract

Herpesviruses are large DNA viruses and include important human and veterinary pathogens. Their genomes can be cloned as bacterial artificial chromosomes (BACs) and genetically engineered in *Escherichia coli* using BAC recombineering methods. While the recombineering methods are efficient, the initial BAC-cloning step remains laborious. To overcome this limitation, we have developed a simple, rapid, and efficient BAC-cloning method based on single-step transformation-associated recombination (STAR) in *Saccharomyces cerevisiae*. The linear viral genome is directly integrated into a vector comprising a yeast centromeric plasmid and a BAC replicon. Following transfer into *E. coli*, the viral genome can be modified using standard BAC recombineering techniques. We demonstrate the speed, fidelity, and broad applicability of STAR by cloning two strains of both rat cytomegalovirus (a betaherpesvirus) and Kaposi’s sarcoma-associated herpesvirus (a gammaherpesvirus). STAR cloning facilitates the functional genetic analysis of herpesviruses and other large DNA viruses and their use as vaccines and therapeutic vectors.

## Motivation

Research in the herpesvirus field has benefited greatly from the highly efficient genetic engineering of viral genomes previously cloned as bacterial artificial chromosomes (BACs) in *E. coli*. However, as the classical BAC-cloning method can be time consuming and inefficient, only a limited number of herpesvirus BACs have been generated so far. To facilitate and accelerate the cloning of herpesvirus genomes, we aimed to develop a method to clone complete genomes in a single step by transformation-associated recombination in yeast. The cloned genomes are then transferred into *E. coli*, where they can be analyzed and genetically modified. The method described here should be generally applicable to herpesviruses and probably also to other large DNA viruses.

## Highlights

• STAR cloning facilitates molecular cloning of herpesvirus genomes as BACs
• Viral genomes are cloned by TAR in yeast and subsequently transferred to *E. coli*
• The genomes of lytically replicating and latent herpesviruses can be cloned
• STAR cloning should also be applicable to other large DNA viruses

## Graphical abstract

**Figure:**
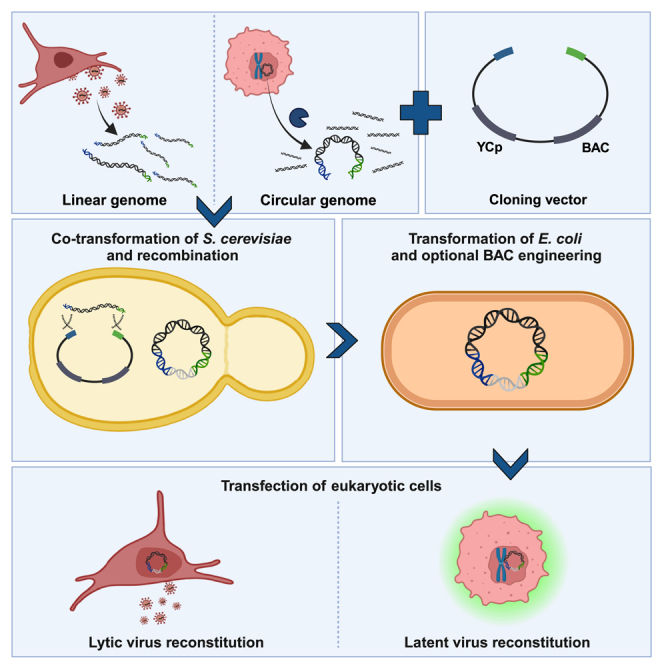

## Teaser

Knickmann et al. present a simple, rapid, and efficient method for cloning of large herpesvirus genomes as bacterial artificial chromosomes (BACs). The method is based on single-step transformation-associated recombination (STAR) in yeast. Following transfer into *E. coli*, the cloned viral genome is amenable to genetic engineering and virus reconstitution.

## Introduction

Herpesviruses cause lifelong persistent infections characterized by periods of latency and sporadic reactivation. Human herpesviruses are responsible for diseases ranging from the acute pustular rashes associated with the alphaherpesviruses herpes simplex virus 1 and 2 (HSV-1 and HSV-2) and varicella-zoster virus (VZV), to the threats posed to those with immature or weakened immune systems by the betaherpesvirus human cytomegalovirus (HCMV), to the lymphoproliferative tumors associated with the gammaherpesvirus Epstein-Barr virus.^1^ Animal herpesviruses similarly represent significant livestock pathogens, and many are also useful models for their human herpesvirus counterpart.^2^^,^^3^ In recent years, several herpesviruses have been explored for their utility as vaccine vectors and oncolytic viral therapeutics.^4^

The ability to clone and maintain large herpesvirus DNA genomes as bacterial artificial chromosomes (BACs) in *E. coli* was introduced over 25 years ago^5^ and represented a sea change in the ability to genetically manipulate herpesviruses.^6^^,^^7^^,^^8^ Having been cloned as a BAC, the herpesvirus genome was then amendable to manipulation using bacteria-based genetic tools, following which reconstitution of the infectious virus could be carried out by transfection of recombinant BACs into susceptible mammalian cells. Over the past two decades, the genetic tools for BAC engineering (recombineering) have been greatly improved and simplified, enabling essentially any change to be quickly generated within a BAC.^9^ In contrast, the initial step of cloning herpesviruses as BACs remains laborious and sometimes problematic, as it relies on integrating the BAC cassette into the herpesvirus genome within virus-infected mammalian host cells. Due to the size limitations for packaging the viral genome within the capsid, this step also commonly requires partial deletion of the viral genome to accommodate the BAC cassette, followed by repair to restore the complete genome. For these reasons, only a small number of herpesvirus genomes have been cloned as BACs.

In an attempt to remove this bottleneck, transformation-associated recombination (TAR) in the yeast *Saccharomyces cerevisiae* has been used to assemble complete herpesvirus genomes from multiple (11–16) overlapping genome fragments.^10^^,^^11^ A hybrid vector consisting of a yeast centromeric plasmid (YCp; a specific type of yeast artificial chromosome [YAC]) and a BAC cassette allowed the subsequent transfer of the cloned genome into *E. coli* for onward recombineering. Although elegant in removing BAC cloning from the confines of the mammalian cell, TAR is laborious and time consuming, as it requires the initial subcloning and verification of numerous subgenomic fragments prior to the assembly of complete viral genomes within the BAC.

Herein, we describe a substantive modification of the earlier TAR-based approach that enables simple and rapid BAC cloning of complete herpesvirus genomes in a single step. We have called this technique—which is, in principle, applicable to all large DNA viruses—single-step TAR (STAR). We demonstrate the feasibility of STAR by cloning the genomes of two rat cytomegalovirus (RCMV) strains and two Kaposi’s sarcoma-associated herpesvirus (KSHV; also known as human herpesvirus 8) strains. These orthoherpesviruses are betaherpesviruses and gammaherpesviruses, respectively, and their genomes differ in size and structure. STAR cloning removes a final hurdle to easy and rapid genetic manipulation of herpesviruses and probably other large DNA viruses.

## Results

### STAR cloning strategy

**Figure 1:**
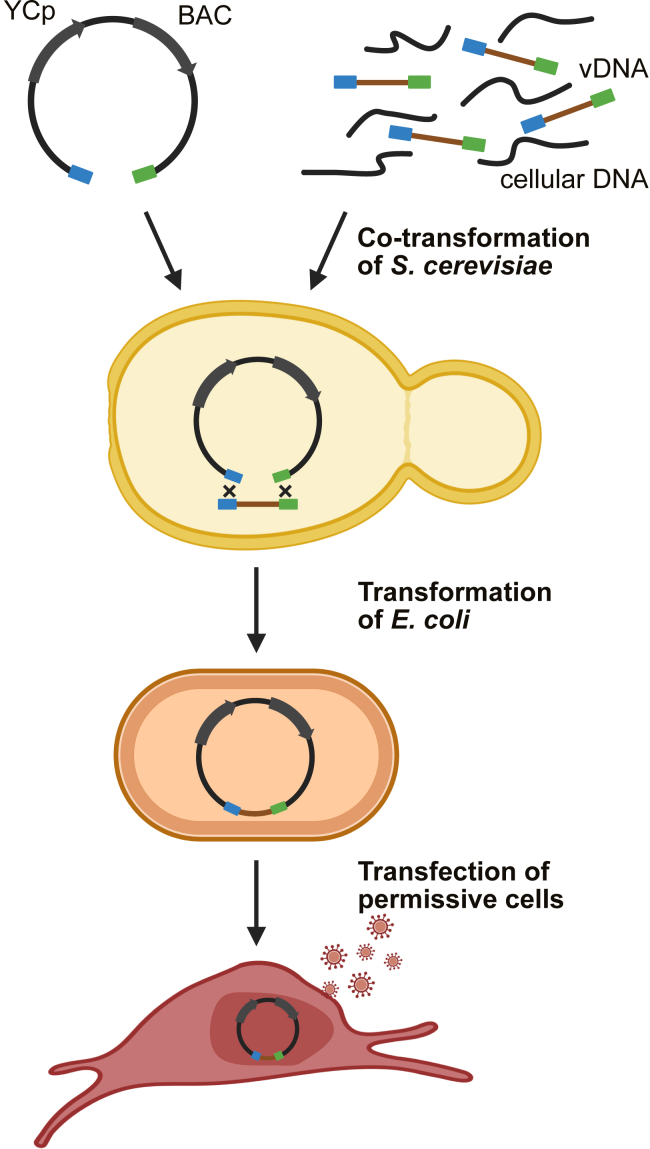
Principle of STAR cloning herpesvirus genomes The linearized cloning vector, which contains yeast centromeric plasmid (YCp) and bacterial artificial chromosome (BAC) replicons, and full-length genomic viral DNA (vDNA) are used to transform yeast (*S. cerevisiae*) spheroplasts. vDNA can be purified or be present within a heterogeneous mixture containing cellular DNA. Homology hooks (≥60 bp, blue and green) recombine with vDNA in yeast to form a YCp. The plasmid DNA isolated from yeast is then used to transform *E. coli*, where it replicates as a BAC. Permissive cells are transfected with purified BAC DNA to reconstitute infectious virus.

In earlier TAR studies of herpesviruses, the genomes of HSV-1 and HCMV were assembled in yeast using 11 (HSV-1) or 16 (HCMV) overlapping subgenomic fragments.^10^^,^^11^ However, the necessary initial cloning of a complete set of overlapping fragments representing the genome as cosmids or BACs was time consuming. We aimed to build on this innovative idea but to greatly simplify and accelerate the process by reducing it to a single step. A schematic outlining STAR cloning is shown in Figure 1. Briefly, we aimed to clone full-length linear viral DNA directly from viral particles or virus-infected cells by TAR using a YCp-BAC vector containing virus-specific homology hooks (Figure 1). Successfully BAC-cloned viral genomes identified by colony PCR would then be transferred to *E. coli* for genetic analysis by BAC digestion and sequencing. Obtained BACs can readily be modified by recombineering. Infectious recombinant viruses would then be reconstituted as usual by transfection of permissive cells with genetically modified BAC DNA (Figure 1).

### STAR cloning of two betaherpesvirus genomes

**Figure 2:**
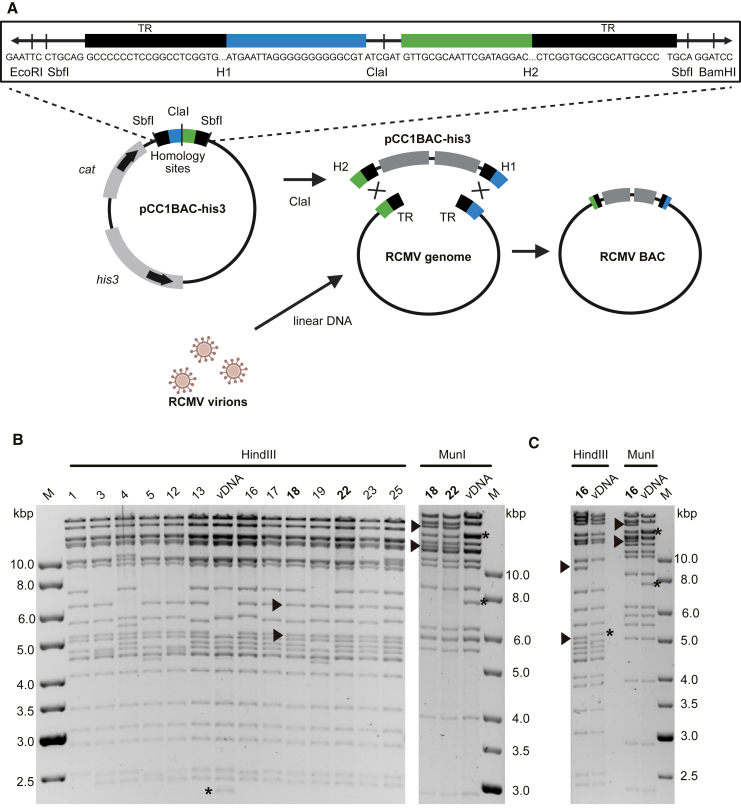
STAR cloning of RCMV genomes isolated from virions (A) Vector construction and STAR cloning strategy. The YCp-BAC vector pCC1BAC-his3 contains *his3* and *cat* markers for selection in yeast and bacteria, respectively. Homology hooks for STAR cloning of RCMV-E comprise the terminal 60 bp at either end of the RCMV-E genome including 30 bp of TRs. Hooks are separated by a ClaI restriction site and flanked by SbfI restriction sites. Linear vDNA was isolated directly from virions in the supernatant of infected REF cells. (B) After transfer from yeast into *E. coli*, BAC DNA was isolated, digested with HindIII, and analyzed by gel electrophoresis. The vDNA isolated from virions served as a control. Differences in the restriction pattern were expected due to the absence of the cloning vector in the vDNA. Two clones, E18 and E22, were also analyzed by MunI digestion and sequencing. (C) Restriction fragment analysis of RCMV-B clone B16 and vDNA. M, DNA size marker. Arrowheads indicate vector-vDNA junction fragments. Asterisks indicate terminal fragments of the linear viral genome that are absent in the circular BAC clones.

To examine the utility and versatility of STAR cloning, we cloned two strains of RCMV, a lytically replicating rat betaherpesvirus that serves as a small-animal model for HCMV infection of humans. Three strains of RCMV (also known as murid herpesvirus 8) have been isolated in England (RCMV-E), Berlin (RCMV-B), and Malaysia.^12^^,^^13^^,^^14^^,^^15^ However, none have yet been cloned as a BAC. To STAR clone the RCMV-E and RCMV-B strains, linear DNA isolated from virions was used. Due to high copy numbers, pure viral DNA could be isolated directly from the infectious cell culture supernatant with minimal cellular DNA contamination. Another advantage was the naturally occurring linearization of the RCMV genome between the two terminal repeats (TRs) at the genome ends during packaging into virions. Thus, no artificial linearization of the genome was needed, and the genomic termini could be used as homology hooks corresponding to the first and last 60 bp sequence of the 203 kbp RCMV genome. RCMV-E-specific hooks were inserted into the YCp-BAC-cloning vector as shown in Figure 2A. Viral DNA was isolated from viral particles collected from the supernatant of RCMV-infected rat embryonic fibroblast (REF) cells. Yeast spheroplasts were co-transformed with 1–2 μg viral DNA and 0.5 μg linearized YCp-BAC vector, plated on SD-SORB/-His agar plates, and incubated for 5 days at 30°C. Yeast colonies were screened by PCR for the presence of RCMV-E DNA using two primer pairs specific to the RCMV genes M47 and M104, respectively. YCp-BAC DNA was extracted from PCR^+^ yeast clones (13 of 30, 43%) and used to transform *E. coli* DH10B by electroporation. RCMV-E YCp-BAC DNA was isolated from bacterial clones by standard BAC purification procedures and analyzed by restriction digestion (Figure 2B). Six of the 13 clones tested (46%) showed the expected restriction pattern.

To verify the sequence integrity, two clones (E18 and E22) were sequenced and compared to the wild-type (WT) RCMV-E genome sequence (GenBank: OP429143). Both clones contained the complete RCMV-E genome without deletions or insertions. A few substitutions and homopolymeric-tract-length polymorphisms were detected when compared to the consensus sequence of the viral DNA used for cloning. Clone E18 contained three polymorphisms in non-coding regions and five substitutions in coding regions, one of which was silent. Clone E22 contained two SNPs in non-coding and two in coding regions (Table S1). Notably, all SNPs detected in the clones were also present as minor variants in the parental RCMV-E pool (Table S1). This indicated that the SNPs most likely represented variants of the viral DNA used for cloning rather than *de novo* mutations introduced by recombination and cloning in yeast or *E. coli*.

To further test the applicability of STAR cloning, we also cloned the RCMV-B genome. Since the genome termini of RCMV-E and RCMV-B are similar but not identical, homology hooks specific to RCMV-B were inserted into the YCp-BAC vector (Table S2). STAR cloning was performed as described for RCMV-E. One clone (B16; Figure 2C) and the viral DNA used for cloning were completely sequenced. No differences were detected.

**Figure 3:**
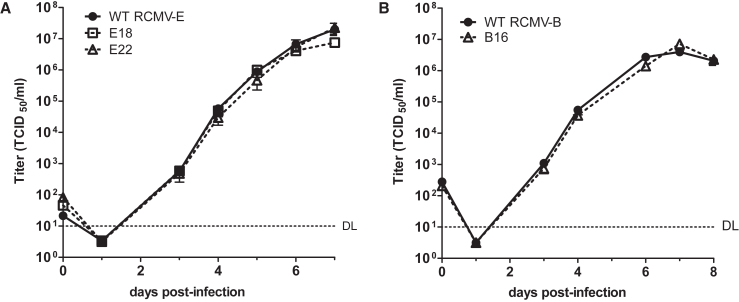
Multi-step RCMV replication kinetics (A) REF cells were infected (MOI 0.05) with the parental RCMV-E- and BAC-derived clones E18 and E22. Viral titers in the supernatants (mean ± SEM of three biological replicates) were determined until day 7 post-infection. (B) Replication kinetics of the parental RCMV-B and clone B16 were analyzed as described above for RCMV-E. DL, detection limit.

RCMVs have a 30 bp direct TR at the genome ends, and the YCp-BAC cloning vector was inserted between them (Figure 2A). This insertion site was selected on the basis of a previous study showing efficient self-directed excision of a BAC vector placed between the genome termini of VZV, an alphaherpesvirus, during virus reconstitution in permissive cells.^16^ However, we also flanked the BAC cassette with SbfI restriction sites (Figure 2A) to enable excision *in vitro* should excision during virus reconstitution be less efficient than anticipated (no SbfI restriction sites are present within the parental RCMV genomes). Transfection of REF cells with BAC-cloned RCMV genomes resulted in rapid and efficient reconstitution of infectious virus using intact circular DNA or SbfI-digested linear DNA. The integrity of the reconstituted viral genomes and the fidelity of cassette excision following transfection of uncut BAC DNA were confirmed by Illumina sequencing. This showed that the insertion of the cloning vector between the RCMV genome termini resulted in reliable cassette excision upon transfection of permissive cells. In addition, the viruses derived from cloned RCMV genomes replicated with similar kinetics to the parental viruses (Figure 3).

### STAR cloning of two gammaherpesvirus genomes

To assess whether the STAR cloning procedure could also be used to clone latent herpesvirus genomes, we used STAR to clone two KSHV strains from primary effusion lymphoma (PEL) cell lines carrying latent KSHV episomes. The PEL cell lines BCBL-1 and HBL-6 contain 15–80 KSHV genome copies per cell.^17^ HBL-6 is a PEL cell line isolated from the same patient as the commonly used BC-1 PEL cell line. Both lines are latently co-infected with Epstein-Barr virus.

**Figure 4:**
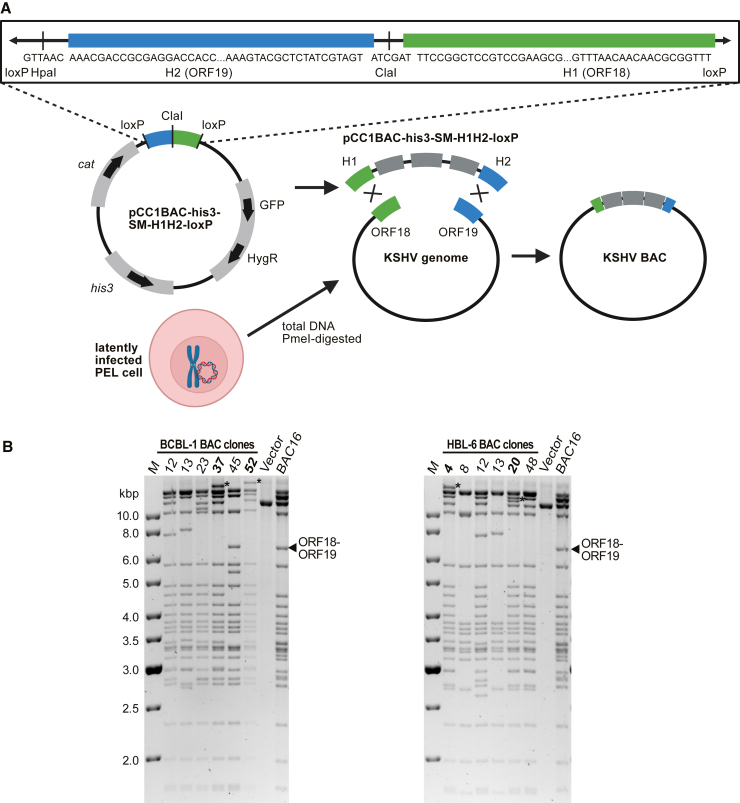
STAR cloning of KSHV genomes from latently infected PEL cells (A) Cloning strategy using a modified pCC1BAC-his3 cloning vector containing a GFP-IRES-Hyg selection marker and KSHV ORF18/19-specific homology hooks (H1 and H2), separated by a ClaI restriction site and flanked by *loxP* sites. (B) After transfer from yeast to *E. coli*, BAC DNA was isolated, digested with BamHI, and analyzed by gel electrophoresis. The JSC-1-derived KSHV BAC16^19^ served as a positive control and the empty cloning vector as a negative control. The fragment containing the TRs is marked by an asterisk. The intact fragment containing ORF18 and ORF19 is marked in JSC-1 BAC16 by an arrowhead. M, DNA size marker.

While KSHV could, in principle, be STAR cloned using the same strategy as for RCMV, there are some critical disadvantages to using this approach. Firstly, the KSHV genome contains more than 18 direct TR copies distributed between the genomic termini.^18^ This would complicate the precise insertion of the vector within the TR region and would put the BAC cassette at risk of excision during productive lytic replication. As KSHV enters latency following infection, the BAC cassette is essential to permit selection of cells carrying the latent KSHV episome. Secondly, the KSHV genome in PEL cells is present as circular episomes, rather than as linear DNA molecules, making it less favorable for recombination-based cloning. To circumvent this, we linearized the circular KSHV genome by digestion with the restriction enzyme PmeI, which cuts at a unique site between open reading frame 18 (ORF18) and ORF19. A further benefit of linearizing the KSHV episomes at this position was the protection of the TR region, which would be prone to deleterious recombination if used as vector insertion site. Consequently, the 60 bp sequences downstream and upstream of the PmeI site were used as homology hooks (Figure 4A). We also modified the pCC1BAC-his3 cloning vector by inserting an EF1a-promoter-GFP-IRES-HygR-pA marker cassette to allow the selection (hygromycin B resistance) and visualization (GFP) of latently infected cells.

For STAR cloning of KSHV, total DNA was isolated from latently infected BCBL-1 and HBL-6 PEL cells. Yeast spheroplasts were co-transformed with 1.5 μg PmeI-digested total DNA and 0.5 μg ClaI-linearized cloning vector. After incubation for 5 days on SD-SORB/-His plates, His^+^ yeast colonies were screened for KSHV ORF8 by PCR. For BCBL-1, 6 of 52 (12%) yeast colonies were ORF8^+^, and for HBL-6, 6 of 29 (21%) yeast colonies were ORF8^+^.

Plasmid DNA was extracted from each PCR^+^ yeast clone and used to transform DH10B *E. coli* by electroporation. BAC DNA was extracted from bacterial clones and analyzed by BamHI restriction digestion and gel electrophoresis. The existing KSHV BAC16, derived from the JSC-1 PEL cell line,^19^ was used for comparison. Four of the BCBL-1-derived BAC clones and four of the HBL-6-derived BAC clones had restriction patterns similar to the BAC16 control (Figure 4B). From the presumptive full-length genome clones, the two with the largest TR bands (BCBL-1 BAC37 and BAC52; HBL-6 BAC4 and BAC20) were analyzed further by complete genome sequencing. Comparison of the parental and BAC-cloned KSHV genomes confirmed that both BCBL-1 BACs (BAC37 and BAC52) and HBL-6 BAC20 had nucleotide sequences identical to KSHV genomes from the parental PEL cell lines. For HBL-6 BAC4, a non-synonymous mutation resulting in an L90W mutation within the ORF24 protein was identified.

**Figure 5:**
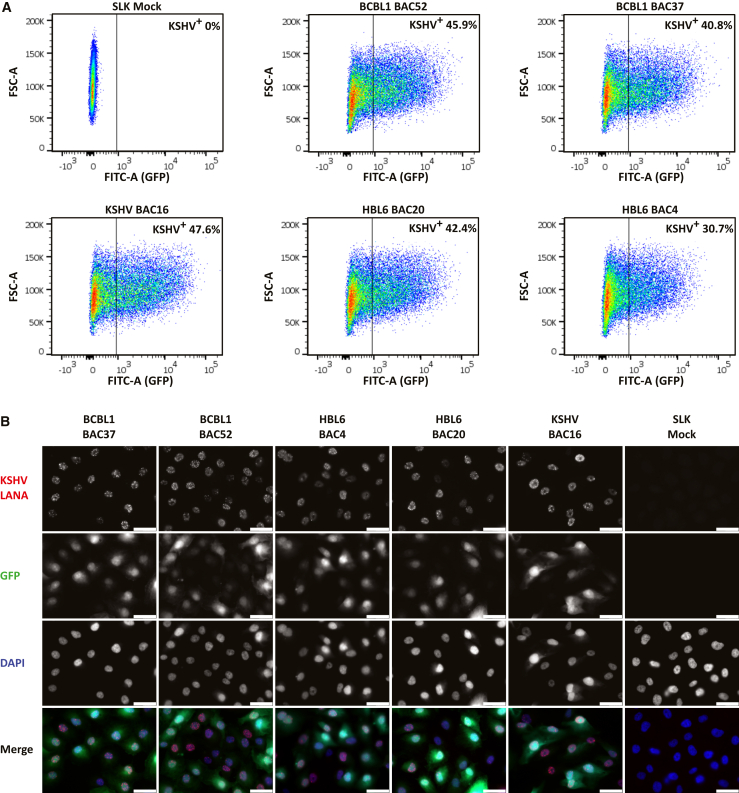
Functional verification of STAR-cloned KSHV BACs (A) Production of infectious virus from iSLK-BAC cells. Virus stocks were prepared by chemical induction of iSLK cells carrying KSHV BACs. SLK cells were infected with 1:2-diluted virus stock, and infected cells expressing GFP were quantified 24 h post-infection by flow cytometry. The gating strategy is shown in Figure S1. (B) Verification of latent infection following BAC-derived virus infection. SLK cells were infected with KSHV BAC-derived virus and stained 24 h post-infection by immunofluorescence for KSHV LANA. Nuclei were counterstained with DAPI. Fluorescence signals were imaged at 63× magnification. Mock- and BAC16-infected SLK cells were used as a negative and positive controls, respectively, in (A) and (B). Scale bar: 45 μm.

To verify the functionality of the sequence-verified KSHV BACs, we reconstituted the BACs in iSLK cells and assessed their ability to generate infectious supernatant (Figure 5A). The hygromycin-resistant and GFP^+^ iSLK cells carrying the KSHV BAC were chemically induced into lytic replication. Cell-free supernatants were concentrated 100× by centrifugation and used to infect SLK cells. KSHV-infected SLK cells were quantified by their green fluorescence via flow cytometry 24 h post-infection (gating strategy in Figure S1). Infection with 1:2-diluted virus stocks resulted in substantial infection rates: 40.8% (BCBL-1 BAC37), 45.9% (BCBL-1 BAC52), 30.7% (HBL-6 BAC4), and 42.4% (HBL-6 BAC20). These infection rates were comparable to the 47.6% infection rate obtained with a stock of the JSC-1-derived BAC16, which was prepared in parallel from BAC16-carrying iSLK cells. Lastly, we confirmed the ability of BAC-derived virus to generate a latent infection in SLK cells. Following infection of SLK cells with BAC-derived virus, latent infection was visualized by immunofluorescence staining for the KSHV latent nuclear antigen (LANA), which tethers the latent KSHV episomes via the TRs to the host chromosomes. For all KSHV BACs, several LANA dots were identified in the nuclei of GFP^+^ SLK cells, indicating that the cells carried several KSHV episomes per cell (Figure 5B).

## Discussion

We describe a rapid and efficient method for BAC cloning of herpesvirus genomes that we have termed STAR cloning. Although STAR cloning is based on earlier TAR-based technology, its use for cloning full-length herpesvirus genomes isolated directly from virions or latently infected cells in a single step significantly reduces the time and effort required for BAC cloning (from multiple months to less than a month). This removes a substantial hurdle, which has limited research for some herpesvirus to only a few BACs. STAR cloning will permit the efficient generation of large BAC libraries exposing more herpesvirus strains to fundamental research and allow the investigation of strain-specific differences within herpesvirus species.

The most commonly used BAC-cloning method involves inserting a BAC cassette into a herpesvirus genome by homologous recombination in virus-permissive mammalian cells. The recombinant viral genome is then isolated as a circular episome from infected cells and transferred to *E. coli* for genetic manipulation. Although this approach is simple and straightforward in principle, it is difficult in practice, resulting in the development of genetic tools for manipulating BAC-cloned viral genomes within *E. coli* far outpacing the methodologies for the essential first step of BAC-cloning viral genomes. This incongruity has several sources. Homologous recombination in mammalian cells is rather inefficient, and the selection and verification of recombinant viruses carrying the BAC vector are time consuming, particularly for slowly replicating herpesviruses (such as cytomegaloviruses) and herpesviruses that are predominantly latent (such as KSHV). Moreover, many herpesviruses do not tolerate overlength genomes, requiring deletion of substantial regions of the viral genome to accommodate the ∼8 kbp BAC cassette consisting of the F-plasmid replicon and selectable markers; these deletions have to be repaired within the BAC to obtain a full-length cloned viral genome.

In contrast to the situation with mammalian cells, homologous recombination in yeast is exceptionally efficient. The first complete viral genome cloned by TAR in yeast was the 36 kbp adenovirus type 2 genome.^20^ Over the years, TAR cloning methods have been improved and utilized for rapid and efficient cloning of large fragments of mammalian, fungal, and bacterial DNA.^21^^,^^22^^,^^23^ More recently, TAR was used to assemble the complete genomes of two herpesviruses (HSV-1 and HCMV) and African swine fever virus (ASFV) as BACs using multiple overlapping subgenomic fragments in just a few steps.^10^^,^^11^^,^^24^^,^^25^ Although this ingenious TAR-based method is far faster than mammalian-cell-based BAC-cloning techniques, it still requires generation of a complete set of overlapping subgenomic fragments, usually cloned as cosmids or BACs. This step requires additional time, especially if multiple strains or variants of a virus are to be cloned. STAR cloning does not require any such cloning and sequence verification of subgenomic fragments. Instead, linear herpesvirus genomes are STAR cloned directly in a single step. STAR cloning may be particularly useful for rapid *de novo* cloning of herpesviruses and for parallel cloning of several strains. As examples, we show here the cloning of two RCMV strains and two KSHV strains. While this article was in preparation, we used this method to clone another four KSHV strains and three newly identified rodent CMVs distantly related to RCMV. As the technology is based on homologous DNA recombination, STAR may also enable the rapid BAC cloning of other important DNA viruses beyond the herpesvirus family.

TAR is most efficient with linear DNA molecules and homologous recombination sites at (or close to) the free ends of these molecules.^21^ As herpesvirus genomes are packaged as linear DNA molecules into viral particles (virions), we cloned virion-derived RCMV genomes by using 60 bp hooks homologous to the viral genome ends. This strategy was successful because these genomes (like those of many rodent cytomegaloviruses) have a single 30 bp TR at each end. Hence, the 60 bp hooks consisted of a 30 bp TR and the adjacent 30 bp unique sequence (Figure 2A). A major advantage of placing the YCp-BAC cloning vector between the genome ends is the fact that the vector is naturally excised during virus reconstitution in mammalian cells. However, this approach is certainly significantly affected in its efficiency if the virus has multiple or long TRs, as the homology hooks of the cloning vector would recombine with each other to generate empty vector clones devoid of viral genomes. Even homology hooks containing short 30 bp TRs (as is the case for RCMVs) can recombine with each other and increase the background of empty vector clones. For viruses with multiple long TRs, a different approach has to be taken. In the examples presented here, we linearized the circular KSHV episomes by restriction digest with the unique cutter PmeI. In cases where unique restriction enzyme cleavage sites are unavailable or impractical, linearization could be achieved by *in vitro* digestion with Cas9 nuclease and a suitable guide RNA.^21^ With the same strategy, it should also be possible to clone genomes of herpesviruses that grow in a highly cell-associated fashion, such as fresh clinical isolates of VZV or HCMV. The concatemeric viral genomes generated during rolling-circle DNA replication could be cut into unit-length genomes and then used for STAR cloning. If removal of the cloning vector is necessary during virus reconstitution, the vector may be flanked by *loxP* sites to allow its removal by *Cre* recombinase. In the case of KSHV, vector removal was not necessary, as KSHV tolerates overlength genomes. However, for other herpesviruses (e.g., CMVs), vector removal is necessary to avoid overlength genomes and compensatory random deletions.

Previous reports have demonstrated that TAR cloning in yeast is remarkably efficient. Clone-positivity rates of 1%–5% were reported with TAR cloning of mammalian genes^23^^,^^26^ and could be increased up to 32% when double-strand breaks were induced near the region to be cloned via restriction digestion or Cas9-mediated cleavage.^27^^,^^28^ Similarly, clone-positivity results of 15%–36% were achieved by linearizing the cloned subgenomic fragments of HCMV and HSV-1, respectively, prior to TAR assembly.^10^^,^^11^ As STAR uses linear virion DNA or linearized episomal DNA, we expected similar efficiencies. For KSHV, we observed clone-positivity rates of 11%–20%, which are comparable to the efficiencies observed previously in other settings. For RCMV, we observed clone-positivity rates of up to 40%, which was probably due to using highly pure virion DNA. Since only a small number of STAR-cloned RCMV and KSHV genomes were completely sequenced, it is not possible to make a precise statement about sequence fidelity. However, the observation that only one cloned KSHV genome contained a single-nucleotide substitution in comparison to the corresponding PEL cell DNA suggests a high fidelity. With such a low error rate, an optional sequence correction via BAC mutagenesis is conceivable and only requires a small additional effort. The size of the KSHV genome restriction fragment containing the TRs varied from clone to clone (Figure 4B). This was to be expected, as TR copy-number variations have previously been described in KSHV and other gammaherpesvirus BACs.^19^^,^^29^

For cloning herpesvirus genomes, STAR has several advantages over the classical BAC-cloning method. It is far faster, as it does not involve the generation of a recombinant virus carrying the BAC vector by homologous recombination in mammalian cells. Ideally, STAR cloning (viral DNA isolation, yeast transformation, PCR screening, transfer to *E. coli*, and restriction digest analysis) can be done within 2 weeks. Moreover, the vector can (at least in some cases) be inserted between the ends of the viral genome, which affords natural and seamless excision. It is also not necessary to delete a non-essential region of the viral genome to accommodate the vector as is often required with the classical BAC method due to capsid genome-packaging constraints (Table 1). STAR cloning is conceptually very similar to TAR assembly of herpesvirus genomes: both rely on TAR in yeast. However, STAR cloning recombines the entire viral genome with the YCp-BAC cloning vector in a single step, whereas TAR assembly requires a set of (usually 11 or more) overlapping subgenomic fragments. If a set of overlapping fragments already exists, TAR assembly is probably as fast and efficient as STAR cloning. However, this is rarely the case even for well-characterized known herpesviruses and never the case for new strains of these viruses or for newly identified herpesviruses. In such cases, subgenomic fragments have to be cloned and sequence verified first, which require additional time and effort. On the other hand, a specific advantage of TAR assembly is the possibility to combine modified fragments or fragments from different viral strains to generate chimeric viruses (Table 1).

**Table 1:** Characteristics of different BAC cloning methods for herpesviruses

	Classical BAC cloning	TAR assembly	STAR cloning
Recombination host	mammalian cell	yeast	yeast
Time requirement	months	weeks^a^	weeks
Cloning of genome fragments required	no	yes	no
Deletion required to accommodate vector	yes (in some cases)	no	no
Vector insertion between genome ends	no	yes	yes
Combinatorial modifications, chimeric strains	no	yes	no
First described in	Messerle et al.^5^	Oldfield et al.^10^	this study

a Provided that cloned overlapping genome fragments exist. Otherwise, additional time is required.

In summary, STAR greatly accelerates and facilitates BAC cloning of herpesvirus genomes. It is particularly useful for *de novo* cloning of herpesviruses for which overlapping cloned subgenomic fragments representing the whole genome are not available. It is also highly applicable to cloning different strains of a herpesvirus and thus facilitating analyses of genomic variability, strain-specific phenotypes, and different growth properties. We anticipate that STAR will be applicable to other large DNA viruses such as poxviruses, asfarviruses, iridoviruses, and baculoviruses. This may be particularly useful for rapidly emerging DNA viruses such as ASFV in pigs, mpox virus in humans, and lumpy skin disease virus in cattle. It may even be possible to STAR clone viral genomes directly from animal or human clinical samples, particularly as TAR assembly has recently been used to clone an ASFV genome from such material.^25^ When available as BAC clones, these viruses can then be genetically engineered for basic research and for development as therapeutic vectors and vaccines.

### Limitations of the method

STAR cloning, like any method, has specific requirements and limitations. An important requirement is viral DNA of high quality and integrity. Herpesvirus genomes are large (125–241 kbp) and thus are susceptible to shearing. To minimize damage, we isolated virion DNA or whole-cell DNA by using a classical procedure involving proteinase K digestion and phenol-chloroform extraction. Vigorous shaking (vortexing) and repeated pipetting were avoided. We also aimed to enrich circular KSHV episomes by using variants of the Hirt extraction protocol.^30^^,^^31^ However, we obtained the best STAR cloning results with whole-cell DNA, without enrichment for episomal viral DNA. *S. cerevisiae* is an excellent host for cloning large DNA molecules. However, it is extremely difficult to isolate useful quantities of yeast circular plasmids from yeast directly. Therefore, a transfer of the YCp-BAC to *E. coli* is necessary for restriction fragment length polymorphism analysis, sequence analysis, and further manipulation by BAC recombineering. To our surprise, the transfer to *E. coli* was in some cases less efficient than anticipated. This observation is consistent with a previous study that reported decreasing transfer efficiencies with larger plasmids, with transfer of YCp-BAC plasmids larger than 250 kbp being very inefficient.^32^ For the transfer of very large plasmids, this publication recommended yeast lysis and proteinase digestion in agarose plugs as a more gentle method compared to the simple alkaline lysis procedure.

## STAR★Methods

### Key resources table

**Table:** 

REAGENT or RESOURCE	SOURCE	IDENTIFIER
**Antibodies**		

Polyclonal rabbit anti-LANA antiserum	Günther et al.^33^	N/A
Goat anti-rabbit-AlexaFluor555	Thermo Fisher Scientific	Cat#A-21428; RRID:AB_2535849

**Bacterial and virus strains**		

RCMV-England	Priscott and Tyrell^12^	N/A
RCMV-Berlin	Geyer et al.^15^	N/A

**Chemicals, peptides, and recombinant proteins**		

Klenow fragment (10 U/μL)	Thermo Fisher Scientific	Cat#EP0051
T4 DNA Ligase (5 U/μL)	Thermo Fisher Scientific	Cat#15224041
FastDigest restriction enzymes (ClaI, BamHI, EcoRI, SbfI, PmeI, BstEII, HpaI, HindIII, MunI)	Thermo Fisher Scientific	Cat# FD0143, FD0045, FD0274, FD1194, FD1344, FD0394, FD0514, FD0504, FD0754
Restriction enzyme SrfI	New England Biolabs	Cat#R0629S
Zymolyase 20T from Arthrobacter luteus	MP Biomedicals	Cat#08320921
SORB/SD/-His with agar	Takara Bio	Cat#630313
SORB/SD/-His broth	Takara Bio	Cat#630312
YPD medium	Carl Roth	Cat#X970.1
GenJet *In Vitro* DNA Transfection Reagent	SignaGen Laboratories	Cat#SL100488
FuGENE 4K Transfection Reagent	Promega	Cat#E5911
Sodium butyrate	Carl Roth	Cat#156-54-7
12-*O*-tetradecanoylphorbol-13-acetate	Sigma Aldrich	Cat#79346
Proteinase K (50 μg/μL)	Thermo Fisher Scientific	Cat#QS0511

**Critical commercial assays**		

NucleoBond Xtra Midi kit	Macherey-Nagel	Cat#11932492; Cat# 12810931
KAPA LTP Library Preparation Kit	Roche Sequencing	Cat#KR0453
Nextera XT DNA Library Prep Kit	Illumina	Cat#FC-131-1096

**Deposited data**		

RCMV-E18 BAC	This study	GenBank: OR343213
RCMV-E22 BAC	This study	GenBank: OR343214
RCMV-B WT	This study	GenBank: OR343211
RCMV-B16 BAC	This study	GenBank: OR343212
KSHV BCBL-1 BAC52	This study	GenBank: OR333977
KSHV HBL-6 BAC20	This study	GenBank: OR333978
KSHV HBL-6 WT	This study	GenBank: OR573937

**Experimental models: Cell lines**		

Rat embryo fibroblasts (REF)	Dr. J. Williams (Johns Hopkins Oncology Center, Baltimore, MD)	Burns et al.^34^
SLK cells	ATCC	RRID:CVCL_9569; Siegal et al.^35^
iSLK cells	ATCC	RRID:CVCL_B6YV; Myoung and Ganem^36^; Stürzl et al.^37^
BCBL-1 PEL	ATCC	RRID:CVCL_0165
HBL-6 PEL	ATCC	RRID:CVCL_4221

**Experimental models: Organisms/strains**		

*E. coli* DH10B	Durfee et al.^38^	https://www.ncbi.nlm.nih.gov/Taxonomy/Browser/wwwtax.cgi?id=316385
S. cerevisiae strain VL6-48N	ATCC	MYA-3666

**Oligonucleotides**		

Primers for PCR screening and homology hook design, see Table S2	This study	N/A

**Recombinant DNA**		

RCMV E18 BAC	This study	N/A
pCC1BAC-his3	Gibson et al.^39^	N/A
pCC1BAC-his3-SM	This study	N/A
RCMV E22 BAC	This study	N/A
RCMV B16 BAC	This study	N/A
KSHV BAC16	Brulois et al.^19^	N/A
KSHV BCBL-1 BAC37	This study	N/A
KSHV BCBL-1 BAC52	This study	N/A
KSHV HBL-6 BAC4	This study	N/A
KSHV HBL-6 BAC20	This study	N/A

**Software and algorithms**		

SPAdes genome assembler v3.15.5	Bankevich et al.^40^	RRID:SCR_000131
GraphPad Prism v5.03	GraphPad Software	RRID:SCR_002798
FlowJo v10.8.1	FlowJo™ Software for Windows, Becton, Dickinson and Company; 2023.	RRID:SCR_008520
QIAGEN CLC Main Workbench 7.9.1	QIAGEN, Aarhus, Denmark	RRID:SCR_000354
Image Lab 2.0.1	Bio-Rad Laboratories, Inc.	RRID:SCR_014210
SnapGene v7.0.2	SnapGene software	RRID:SCR_015052
Affinity Designer v1.10.6.1665	Serif (Europe) Ltd (1987).	RRID:SCR_016952
bcl2fastq v2.20.0.422	Illumina	RRID:SCR_015058
minimap2 v2.14-r883	Li^41^	RRID:SCR_018550
sambamba v0.7.1	Tarasov et al.^42^	RRID:SCR_024328
IGV v2.12.2	Robinson et al.^43^	RRID:SCR_011793

### Resource availability

#### Lead contact

Further information and requests for resources and reagents should be directed to and will be fulfilled by the lead contact, Wolfram Brune (wolfram.brune@leibniz-liv.de).

#### Materials availability

All unique/stable reagents generated in this study are available from the lead contact without restriction for use in academic research. For commercial research, they are available with a completed Materials Transfer Agreement.

#### Data and code availability

(1) Viral genome and BAC sequences generated in this study have been deposited at GenBank and are publicly available as of the date of publication. Accession numbers are listed in the key resources table. Raw data (incl. microscopy, electrophoresis, FACS) reported in this paper will be shared by the lead contact upon request.
(2) This study does not report original code.
(3) Any additional information required to reanalyze the data reported in this paper is available from the lead contact upon request.

### Experimental model and study participant details

For STAR cloning, the highly-transformable and HIS3-deficient S. cerevisiae strain VL6-48N (MATa, his3-Δ200, trp1-Δ1, ura3–52, lys2, ade2–101, met14)^44^ was used.

RCMV strains England and Berlin (RCMV-E and RCMV-B) have been described previously.^12^^,^^13^^,^^15^ RCMV was propagated in REF cells (sequence verified, male),^34^ which were cultured in complete Dulbecco’s modified Eagle’s medium (DMEM, Gibco) containing 10% (v/v) fetal calf serum (FCS), 100 IU/mL penicillin and 100 μg/mL streptomycin (Sigma) at 37°C and 5% CO_2_ atmosphere.

KSHV-containing PEL cell lines BCBL-1 and HBL-6 (both from male patients) were cultured as suspension cultures in RPMI 1640 medium (Gibco) supplemented with 10% (BCBL-1) or 20% FCS (HBL-6). KSHV BACs were reconstituted in iSLK cells,^36^^,^^37^ a KSHV producer cell line encoding a doxycycline-inducible KSHV replication and transcription activator (RTA) gene. iSLK cells were cultured in complete DMEM supplemented with 250 μg/mL G418 (Gibco) and 1 μg/mL puromycin (Sigma). iSLK cells containing a KSHV BAC were additionally selected using 400 μg/mL hygromycin B (Roth). BAC-derived KSHV infectivity was verified in SLK cells.^35^

### Method details

#### Plasmid preparation

All STAR cloning vectors were based on the previously described CopyControl pCC1BAC-his3 plasmid.^45^^,^^39^ This vector contains a bacterial F plasmid-derived replicon (BAC cassette) and a YCp replicon for propagation in bacteria and yeast, respectively. It also contains a chloramphenicol acetyltransferase (*cat*) and a *his3* gene for selection in *E. coli* and *S. cerevisiae*, respectively.

For cloning of RCMV strains from linear virion DNA, two 60 bp homology hooks separated by a ClaI restriction site were inserted into pCC1BAC-his3. Two single-stranded oligonucleotides (RCMV_E_H1 and RCMV_E_H2; Table S2) were combined in annealing buffer (100 mM NaCl, 10 mM Tris-HCl pH 7.4), incubated at 95°C for 2 min, and cooled down to RT. A complete double-stranded oligonucleotide was obtained by filling in with Klenow DNA polymerase (Thermo Fisher). After heat-inactivation, the oligonucleotide was digested with EcoRI and BamHI and inserted into the pCC1BAC-his3 vector. The homology hooks were identical to the first and last 60 bp of the linear RCMV genome, which consist of a 30 bp direct TR followed by a 30 bp unique sequence. Hooks were flanked by SbfI restriction sites for an optional excision of the pCC1BAC-his3 vector before virus reconstitution. A ClaI restriction site was placed between the hooks for linearization of the vector and separation of the two hooks prior to STAR cloning.

For cloning of episomal KSHV DNA from latently infected cells, the pCC1BAC-his3 vector was modified to facilitate the selection of latently infected cells. The eukaryotic selection marker (SM) cassette (*lox*P-EF1a-promoter-GFP-IRES-HygR-pA) from KSHV BAC16^19^ was PCR-amplified (KSHV_SM_cassette_fwd and KSHV_SM_cassette_rev; Table S2) and inserted between the BstEII and HpaI sites of the pCC1BAC-his3 vector, resulting in pCC1BAC-his3-SM. The homology hooks represented the sequences 60 bp upstream and downstream of the unique PmeI restriction site between ORF18 and ORF19 within the KSHV genome (GenBank: HQ404500, nt 32717). A ClaI restriction site was placed between the hooks for hook separation. To allow for the optional removal of the BAC cassette following BAC cloning, an additional *loxP* site was added downstream of the homology hooks. A double-stranded oligonucleotide containing hook2-ClaI-hook1-*lox*P was synthesized as a gBlock (Integrated DNA Technologies; Table S2) and inserted between the HpaI and SrfI restriction sites of pCC1BAC-his3-SM, resulting in the cloning vector pCC1BAC-his3-SM-H2H1-*lox*P.

#### DNA isolation

RCMV DNA was isolated from virions concentrated from the supernatant of RCMV-infected REF cells. KSHV DNA was isolated as total DNA from latently infected PEL cells. Either 1x10^6^ PEL cells washed with PBS (Sigma-Aldrich) or 100–200 μL 1000× concentrated RCMV-containing supernatant were resuspended in 500 μL buffer I (75 mM EDTA (Serva), 75 mM NaCl). 500 μL buffer II (100 mM Tris-HCl pH 8.0 (Sigma-Aldrich), 10 mM EDTA, 10 mM NaCl, 1% (w/v) SDS (Roth)) and 1 mg/mL RNase A (Roth) were added. The mixture was incubated for 20 min at room temperature (RT), supplemented with proteinase K (0.2 mg/mL; Thermo Fisher), and incubated for 4 h at 56°C.

DNA was isolated by PCI (phenol:chloroform:isoamyl alcohol; 25:24:1 [v/v]; Roth) extraction. Briefly, 500 μL of PCI was added to the proteinase K-digested lysates. After gentle mixing for 5 min, phases were separated by centrifugation at 12,000 × *g* for 5 min at RT. The upper (aqueous) phase was extracted once more with PCI, mixed with 500 μL chloroform (Roth), and centrifuged at 12,000 × *g* for 5 min at RT. The upper phase was transferred to a new tube and DNA was precipitated by adding 1 vol. isopropanol and 0.1 vol. 3 M sodium acetate (pH 5.6), followed by incubation for 20 min at −80°C. DNA was pelleted by centrifugation at 22,000 × *g* for 1 h at 4°C. The DNA pellet was washed twice with 70% (v/v) ethanol, dried, and carefully resuspended in 10 mM Tris-HCl pH 8.0. DNA concentrations were determined using a NanoDrop spectrophotometer (Thermo Fisher).

As recombination in yeast is especially efficient close to dsDNA breaks, the circular KSHV genomes (within the PEL-derived whole-cell DNA) were linearized at the desired BAC integration site using the unique cutter PmeI. For this 1.5 μg total PEL-derived DNA was digested with 3 μL FastDigest PmeI (Thermo Fisher) for 45 min at 37°C followed by thermal inactivation for 10 min at 65°C.

#### STAR cloning of viral DNA in yeast

Transformation of *S. cerevisiae* strain VL6-48N was performed as described previously.^46^ The yeast was streaked onto YEPD plates (2% (w/v) D-glucose, 1% (w/v) bacto yeast extract, 2% (w/v) bacto peptone, 2% (w/v) bacto agar (Roth)) and incubated for 3 days at 30°C. One day before STAR cloning, 100 mL YEPD medium was inoculated with one yeast colony from the YEPD plate. The liquid culture was placed in a shaking incubator (230 rpm) at 30°C until the OD_660_ reached 2.0–2.5, cooled on ice for 5 min and pelleted by centrifugation at 1,200 × *g* for 5 min at 4°C. Yeast cells were then washed in 40 mL 1 M sorbitol and resuspended in 40 mL SPE buffer (1 M sorbitol (Sigma-Aldrich), 10 mM sodium phosphate (Sigma-Aldrich), 10 mM Na_2_EDTA (Serva), pH 7.5). Yeast spheroplasts were prepared by incubation at 30°C and 70 rpm with 40 μL Zymolyase 20T (10 mg/mL; MP Biomedicals) in the presence of 80 μL 2-mercaptoethanol (Roth). The spheroplast suspension was, separately, diluted 1:10 in 1 M sorbitol and 2% (w/v) SDS solution, and monitored at OD_660_. Zymolyase digestion was stopped when the SDS:sorbitol OD_660_ reached 1:3 to 1:5. Spheroplasts were pelleted as before and carefully washed twice in 50 mL of 1 M sorbitol. The transformation-ready spheroplasts were resuspended in 2 mL STC buffer (1 M sorbitol, 10 mM Tris-HCl, 10 mM CaCl_2_, pH 7.5).

For transformation, 200 μL spheroplast suspension was mixed with 1–2 μg of linear target DNA and 0.5 μg of linearized STAR cloning vector. The suspension was incubated for 10 min at RT. An aliquot of 800 μL PEG8000 solution (20% (w/v) PEG8000, 10 mM CaCl_2_, 10 mM Tris-HCl, pH 7.5) was added, mixed by inversion, and incubated for 15 min at RT. Spheroplasts were pelleted as before and carefully resuspended in 800 μL SOS solution (1 M sorbitol, 65 mM CaCl_2_, 0.25% (w/v) yeast extract, 0.5% (w/v) peptone (Roth)). The spheroplast suspension was incubated for 40 min at 30°C, mixed with 7 mL SD-SORB-TOP/-His agar (1 M sorbitol, 2% (w/v) D-glucose, 0.17% (w/v) yeast nitrogen base, 0.5% (w/v) (NH_4_)_2_SO_4_, 3% (w/v) bacto agar; cooled to 55°C) and immediately poured onto an SD-SORB/-His agar plate (1 M sorbitol, 2% (w/v) D-glucose, 0.17% (w/v) yeast nitrogen base, 0.5% (w/v) (NH_4_)_2_SO_4_, 2% (w/v) bacto agar; Takara Bio). Plates were incubated for 5 days at 30°C in a humidified incubator.

On day 5 after transformation, yeast drop colonies were prepared by resuspending yeast colonies in 7 μL SD-SORB/-His medium, spotting them onto an SD-SORB/-His plate, and incubating the plate for 2 days at 30°C. Colony PCR was used to screen for the presence of the viral genome. Template DNA was prepared by resuspending half of a yeast drop colony in 50 μL yeast miniprep buffer I (10% (w/v) sucrose (Roth), 50 mM Tris-HCL pH 8.0, 10 mM EDTA, 100 μg/mL RNase A) containing 0.2 mg/mL Zymolyase 20T and 0.1% (v/v) 2-mercaptoethanol. The suspension was incubated for 2 h at 30°C with shaking every 15 min. The Zymolyase was inactivated at 98°C for 5 min, and template DNA-containing solution was used for PCR or stored at −20°C. For colony PCR, 25 μL reactions were prepared using 1.5 μL template solution. Primers specific for the cloned virus were used (Table S2).

#### Transfer of cloned viral genomes from yeast to *E. coli*

DNA from PCR-positive yeast colonies was isolated using a modified alkaline lysis protocol. Briefly, 5 mL SD-SORB/-His medium was inoculated with the remaining half of the drop colony and incubated for 30 h at 30°C in a shaking incubator. The 4 mL yeast cultures were pelleted by centrifugation at 1500 × *g* for 5 min at RT and resuspended in 200 μL of yeast miniprep buffer I containing 0.5 mg/mL Zymolyase 20T and 2-mercaptoethanol (1:500). The suspension was incubated at 37°C for 2 h with shaking every 15 min 400 μL yeast miniprep buffer II (0.2 M NaOH (Merck), 1% (w/v) SDS) was added, and the cells were mixed thoroughly and incubated for 4 min at RT. 300 μL of yeast miniprep solution III (3 M potassium acetate (Merck), pH 8.0) was added and the solution was cleared by centrifugation at 12,000 × g for 10 min at 4°C. The supernatant was combined with 0.8 vol. of isopropanol and centrifuged again at 16,000 × *g* for 1 h at 4°C. The supernatant was removed, and the DNA pellet was washed twice with 500 μL 70% (v/v) ethanol. Finally, the DNA was resuspended in 50 μL 10 mM Tris-HCl, pH 8.0. For transfer of the cloned viral genomes into *E. coli*, 5–10 μL of the yeast-derived DNA was used to transform *E. coli* strain DH10B by electroporation using a BioRad Gene Pulser II. Bacteria were plated on chloramphenicol-containing LB agar plates and incubated overnight at 37°C.

#### BAC purification and sequencing

BAC DNA was isolated from 500 mL LB culture using a NucleoBond Xtra Midi kit (Macherey-Nagel) according to the manufacturer’s protocol for low-copy plasmids. For restriction fragment analysis, approx. 1 μg BAC DNA was digested with restriction enzymes (e.g., HindIII or MunI for RCMV BACs, BamHI for KSHV BACs) and separated by electrophoresis on a 0.6% (w/v) agarose/0.5×TBE (45 mM Tris, 45 mM boric acid (Roth), 1 mM Na_2_EDTA, pH 8.0) gel.

BAC-cloned viral genomes were sequence-verified by Illumina sequencing and compared to the parental viral genome sequence. For sequencing of RCMV England samples, approximately 100 ng of DNA was sheared in an S220 focused-ultrasonicator (Covaris) to fragments of approximately 450 bp. Sequencing libraries were produced by conducting seven PCR cycles using a KAPA LTP library preparation kit (Roche Sequencing and Life Science) with NEBNext multiplex oligos for Illumina (New England Biolabs). For all KSHV and RCMV Berlin samples, the libraries were prepared from 1 ng DNA using the Nextera XT DNA Library Prep Kit (Illumina) according to the manufacturer’s protocol. The libraries were analyzed on NextSeq 500 and NextSeq 550 instruments (Illumina) generating datasets of 150 bp paired end reads. The sequences were assessed by *de novo* assembly using SPAdes genome assembler v3.15.5^40^ and alignment with the respective reference genomes using Bowtie2.^40^ The sequences of parental strains as well as the BAC sequences have been deposited in GenBank (accession numbers are in the key resources table).

#### RCMV reconstitution from cloned genomes

To reconstitute RCMV from BAC DNA, REF cells were transfected with 4 μg purified BAC DNA using GenJet (SignaGen) transfection reagent. Briefly, for each transfection 5×10^5^ REF cells were harvested and pelleted by centrifugation at 150 × *g* for 10 min. Meanwhile, 4 μg of purified BAC DNA was diluted in 100 μL serum-free DMEM and mixed with another 100 μL DMEM containing 6 μL GeneJet transfection reagent. The transfection mix was incubated for 10–15 min at RT before being used for resuspension of the REF cell pellet. The cell suspension was incubated for 20 min at 37°C. Afterward, 2 mL of pre-warmed DMEM with 10% FCS was added to the cells and plated in 6-well dishes. The medium was changed after 24 h. An optional digest of RCMV BAC DNA with SbfI could be performed prior to transfection in order to release the pCC1BAC-his3 backbone.

#### Generation of latently KSHV-infected cells and production of infectious KSHV

KSHV BACs were reconstituted in iSLK cells by transfection with FuGENE HD (Promega). KSHV BAC DNA was isolated from a 4 mL bacterial culture and resuspended in 40 μL water. iSLK cells were seeded at 9×10^4^ cells per well in a 6-well plate and grown to approx. 70% confluency. 30 min prior to transfection, the medium was changed to OptiMEM (Gibco). The transfection mixture was prepared by combining 10% of the prepared BAC DNA and 5 μL FuGENE HD in 85 μL OptiMEM. The mixture was incubated for 10 min at RT and then distributed onto the cells. After 4 h, 10% FCS was added to the wells. The medium was changed to DMEM with 10% FCS 24 h post-transfection, and selection with 400 μg/mL hygromycin B was started when the cells reached confluency. Transfected cells were cultured for 2–3 weeks until GFP-positive colonies could be identified (iSLK-BAC cell lines).

KSHV BAC-derived virus stocks were generated from hygromycin-selected iSLK-BAC cultures. Lytic replication of KSHV was stimulated by doxycycline-induced RTA expression and treatment with sodium butyrate (Roth) and 12-*O*-tetradecanoylphorbol-13-acetate (TPA, Sigma-Aldrich). Briefly, iSLK-BAC cells were grown to 40% confluency and induced with 1.0 mM sodium butyrate, 20 ng/mL TPA and 1.0 μg/mL doxycycline (Sigma-Aldrich). After 48 h of induction, the induction medium was replaced by DMEM with 10% (v/v) FCS. 96 h after medium change, the supernatant was collected and cleared of cellular debris by centrifugation at 950 × *g* for 10 min at 4°C. It was then filtered using a 45 μm filter unit, and the virus was pelleted by centrifugation at 25,000 × *g* for 3 h at 4°C. The virus pellet was resuspended in DMEM with 0.5% FCS in 1% of the original supernatant volume and stored at −80°C.

#### RCMV stocks and replication kinetics

To compare virus replication kinetics of WT RCMV and reconstituted BAC clones, virus stocks were generated and titrated.^47^ Briefly, REF cells were infected at a multiplicity of infection (MOI) of 0.05. After 5 to 7 days, virus-containing supernatant was centrifuged at 6,000 × *g* for 10 min to remove cell debris and concentrated 1,000× by centrifugation at 27,000 × *g* for 3 h and resuspension in a smaller volume of DMEM. Stocks were aliquoted and stored at −80°C. Titration of virus stocks was performed using the 50% tissue culture infectious dose (TCID50) method.^48^ Briefly, 1.5×10^3^ REF cells per well were seeded in 96-well plates. On the following day, 100 μL of virus stock was added to each well (eight different 10-fold dilutions were applied to 12 wells each). Cells were incubated for 7 days at 37°C and 5% CO_2_ atmosphere. Each well was screened manually and CPE-positive wells were counted for each dilution. These numbers were used to determine the TCID_50_ titer using Spearman-Kärber formula.

For replication kinetics analyses, 1×10^4^ REF cells per well were seeded in 6-well plates. On the following day, cells were infected at an MOI of 0.05. The inoculum was removed 4 h post-infection, cells were washed twice with PBS, and fresh medium was added. Supernatants were collected daily for 8 days and stored at −80°C. Virus titers were determined by titration of the supernatants using the TCID_50_ method.

#### Measurement of KSHV infectivity

As KSHV enters latency following infection, BAC-derived KSHV stocks were titrated by quantification of GFP-positive cells following infection with cell-free virus supernatant as described previously.^19^ SLK cells (1×10^5^ per well) were seeded in 6-well plates 12 h prior to infection. Cells were infected with a 1:2 diluted virus stock, followed by centrifugal enhancement at 2,000 × *g* for 30 min at 37°C. At 1 h post-inoculation, cells were washed with PBS and cultured in DMEM with 10% FCS. At 24 h post-inoculation, cells were detached by trypsinization, washed in PBS, and fixed in 2% (w/v) paraformaldehyde (Roth) for 5 min. Fixed cells were pelleted and resuspended in 800 μL PBS containing 10% FCS. The percentage of GFP-expressing cells was quantified for 20,000 cell samples with a FACSCanto II (Beckton Dickinson) flow cytometer.

#### Immunofluorescence

The KSHV latent nuclear antigen (LANA) was detected by immunfluorescence staining. SLK cells (2.5×10^4^ cells per well) were seeded in 8-well μ-slides (Ibidi) one day prior to infection. Cells were infected with 50 μL virus stock for 6 h and washed with PBS. Infected cells were cultured until 24 h post-infection and fixed with 4% (w/v) paraformaldehyde for 15 min at RT. Fixed cells were washed with PBS, incubated in 50 mM (w/v) NH_4_Cl for 20 min at RT, washed again with PBS, permeabilized with PBS/2% (v/v) Triton X-100 (Roth) for 20 min at RT, and blocked in TBS with 5% (w/v) glycine (Serva), 5% (w/v) BSA (Sigma-Aldrich), 0.05% (v/v) Tween (Roth), 0.05% (w/v) sodium azide (Roth) for 30 min at RT. Cells were stained using a polyclonal rabbit anti-LANA antiserum^33^ for 1 h at RT, followed by three washes with PBS and incubation with a goat anti-rabbit-AlexaFluor555 (Thermo Fisher) secondary antibody and 4′,6-diamidine-2′-phenylindole dihydrochloride (DAPI, Sigma-Aldrich) for 30 min at RT. Fluorescence images were acquired by using a Nikon A1+ confocal laser scanning microscope.

### Quantification and statistical analysis

Viral titers from replication kinetics of RCMV were displayed using GraphPad Prism v5.03. Each dot represented the mean of three biological replicates with error bars representing the SEM. No statistical tests were used. For FACS analysis, at least 20,000 events were measured per sample.

## Supplemental information

Document S1. Figure S1 and Tables S1 and S2

Document S2. Article plus supplemental information
