# Supplementary material for: A simple method for rapid cloning of complete herpesvirus genomes
Source: Cell Rep Methods. 2024 Jan 23;4(2):100696. doi: 10.1016/j.crmeth.2024.100696 (PMC10921015; doi:10.1016/j.crmeth.2024.100696)
Supplement: Document S1. Figure S1 and Tables S1 and S2 [file mmc1.pdf]

Cell Reports Methods, Volume *4*

Supplemental information

A simple method for rapid cloning

# of complete herpesvirus genomes

Jan Knickmann, Laura Staliunaite, Olha Puhach, Eleonore Ostermann, Thomas Günther, Jenna Nichols, Michael A. Jarvis, Sebastian Voigt, Adam Grundhoff, Andrew J. Davison, and Wolfram Brune

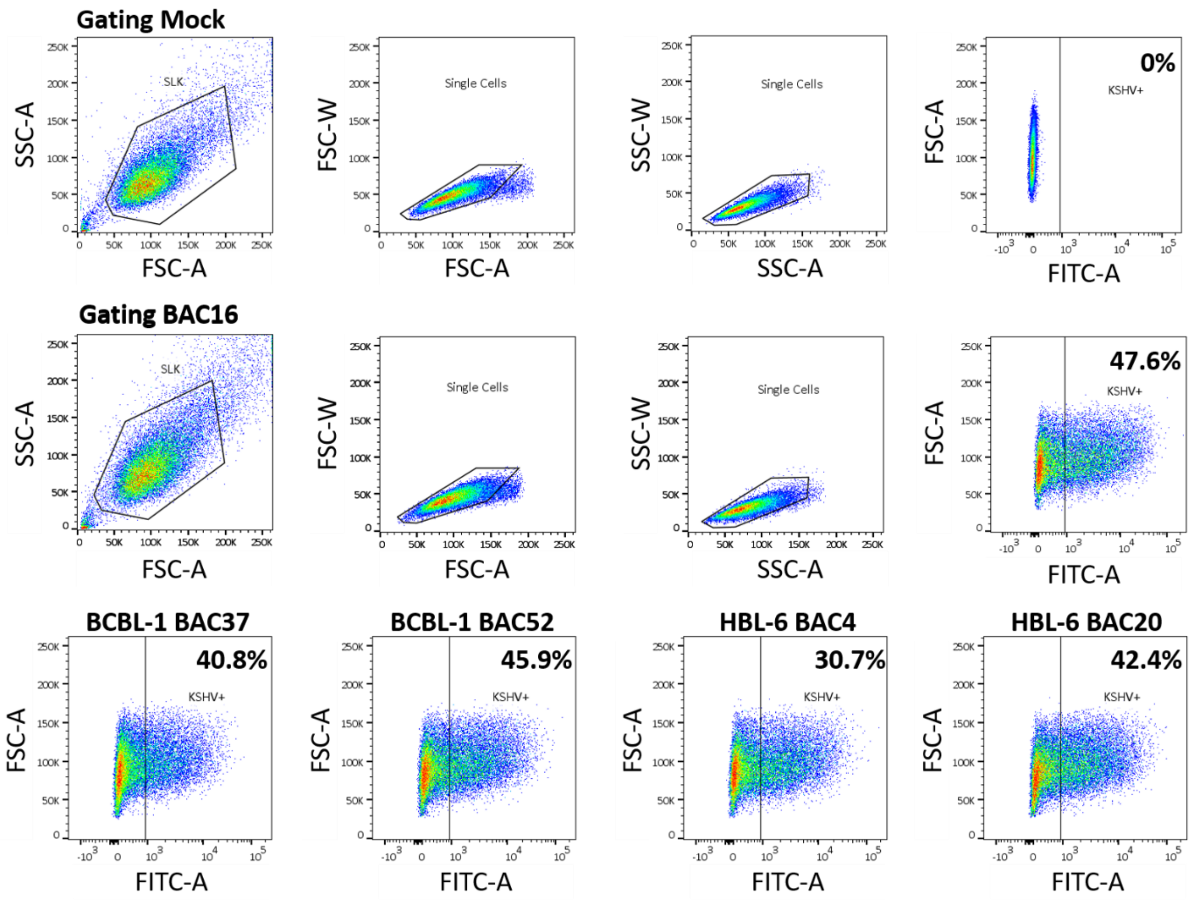

Figure S1. Gating strategy and quantification of infection rates following infection of iLSK cells with BAC-derived KSHV by FACS analysis, related to Figure 5. Virus stocks were prepared from the chosen BACs as described in the materials and methods section. SLK cells were infected with a 1:2 diluted virus stock and the *de novo* infected cells quantified 24 hours post infection by FACS sorting for GFP-expressing cells. The gating strategy for SLK cells, single cells, and GFP-expressing/KSHV-infected cells is shown. The quantification of infection rates for the four different KSHV BACs compared to KSHV BAC16 and mock-infected SLK cells is shown.

Variant	Location	Gene	Type	Effect	RCMV-E WT Consensus	Variant	Reads WT	Reads Variant
E18	48	NC	tract	-	TTAGGGGGGGGGGCGTG	TTAGGGGGGGGGGGCGTG	3408	1636
E22	46.837	M47	SNP	R > Q	CTGACCTACGATTTCCGAT	CTGACCTACAATTTCCGAT	6904	124
E18	51.581	M48	SNP	K > N	CTATAAAAAACTCGAAAAT	CTATAAAAACCTCGAAAAT	6321	693
E22	64.497	M55	SNP	K > N	CTTCAAGATCTTGACACTA	CTTCAAGATATTGACACTA	4582	2066
E18	65.941	M55	SNP	T > I	AAATCATGAGTCTCATTCA	AAATCATGAATCTCATTCA	3529	652
E18	81.435	M71	SNP	silent	TTCCGTACCCGACACCGTT	TTCCGTACCGGACACCGTT	7503	2
E22	131.267	NC	tract	-	CTACAAAAAAAAAAAATCC	CTACAAAAAAAAAAATCC	2558	2011
E22	150.125	NC	SNP	-	TGCAGTTTCGGTGTCCTTA	TGCAGTTTCAGTGTCCTTA	5134	2424
E18	162.313	m139	SNP	G > S	CGTGCGAACCCAGGATCAC	CGTGCGAACTCAGGATCAC	6959	14
E18	173.262	NC	tract	-	TCAAAAAAAAAAAGACAAA	TCAAAAAAAAAAAAGACAAA	3819	1878
E18	200.888	e173	SNP	D > G	GGAGCATAGACTTTTCGGT	GGAGCATAGGCTTTTCGGT	5303	454
E18	201.984	NC	SNP	-	CCCTCACCCAACCTCTAAC	CCCTCACCCGACCTCTAAC	2484	79

Table S1. Sequence analysis of RCMV-E clones, related to Figure 2. RCMV-E clones E18 and E22 and the parental WT virus were analyzed by Illumina sequencing. Differences to the WT RCMV consensus sequence are listed and their presence in the clones are underlined. NC, non-coding.

Oligonucleotide name	Sequence
KSHV_SM_cassette_fwd	5'-TATAGGTTACCCCATAGAGCCCACCGCAT-3'
KSHV_SM_cassette_rev	5'-AATTGTTAACATAACTTCGTATAATGTATGCTATACG-3'
KSHV_hook2-ClaI-hook1-*lox*P (gBlock, IDT)	5'-/Phos/AACAAACGACCGCGAGGACCACCGGCAGGCAGCCAAGAACCATAAAGTACGCTCTATCGTAGTATCGATTTCCGGCTCCGTCCGAAGCGCAGCTGGCCTACCTTGGTGCGTTTAACAACAACGCGGTTTATAACTTCGTATAGCATACATTATACGAAGTTATGCC-3'
RCMV_fwd_1	5'-CAGAAGCGAACTAACGGAGGC-3'
RCMV_rev_1	5'-TGGAAGAGGCAAGGGATCGG-3'
RCMV_fwd_2	5'-AGACGTTGGTTGTGGTATCGG-3'
RCMV_rev_2	5'-CTTCGATCGGCATTGTCCCC-3'
KSHV_ORF8_Fwd	5'-AACACCACTGACATATCCAC-3'
KSHV_ORF8_Rev	5'-AGATCATCCGCCTTCTGC-3'
RCMV_E_H1	5'-TTAAGAATTCCTGCAGGCCCCCCTCCGGCCTCGGTGCGCGCATTGCCCCGGGGGGGATGAATTAGGGGGGGGGGCGTATCGATGTTGCGCAATTTCGATA-3'
RCMV_E_H2	5'-ATATGGATCCTGCAGGGCAATGCGCGCACCGAGGCCGGAGGGGGGGAGAAAAAGTCCTATCGAAATTGCGCAACATCGATACGCCCCCCCCCCTAATTCA-3'
RCMV_B_H1	5'-TTAAGAATTCCTGCAGGCCCCCCTCCGGCCTCGGTGCGCGCATTGCCCCGGGGGGGATGATTTAGGGGGGGGGCGTGGTATCGATGTTGCGCAATTTCGATA-3'
RCMV_B_H2	5'-ATATGGATCCTGCAGGGCAATGCGCGCACCGAGGCCGGAGGGGGGGAGAAAAAGTCCTATCGAAATTGCGCAACATCGATACCACGCCCCCCCCCTAAATCA-3'

Table S2. List of oligonucleotides and gBlocks used in this study, related to STAR Methods.
